# Supplementary figures and images for: Maintenance of Methyl-Esterified Pectin Level in Pollen Mother-Cell Stages Is Required for Microspore Development
Source: Plants (Basel). 2023 Apr 20;12(8):1717. doi: 10.3390/plants12081717 (PMC10142773; doi:10.3390/plants12081717)

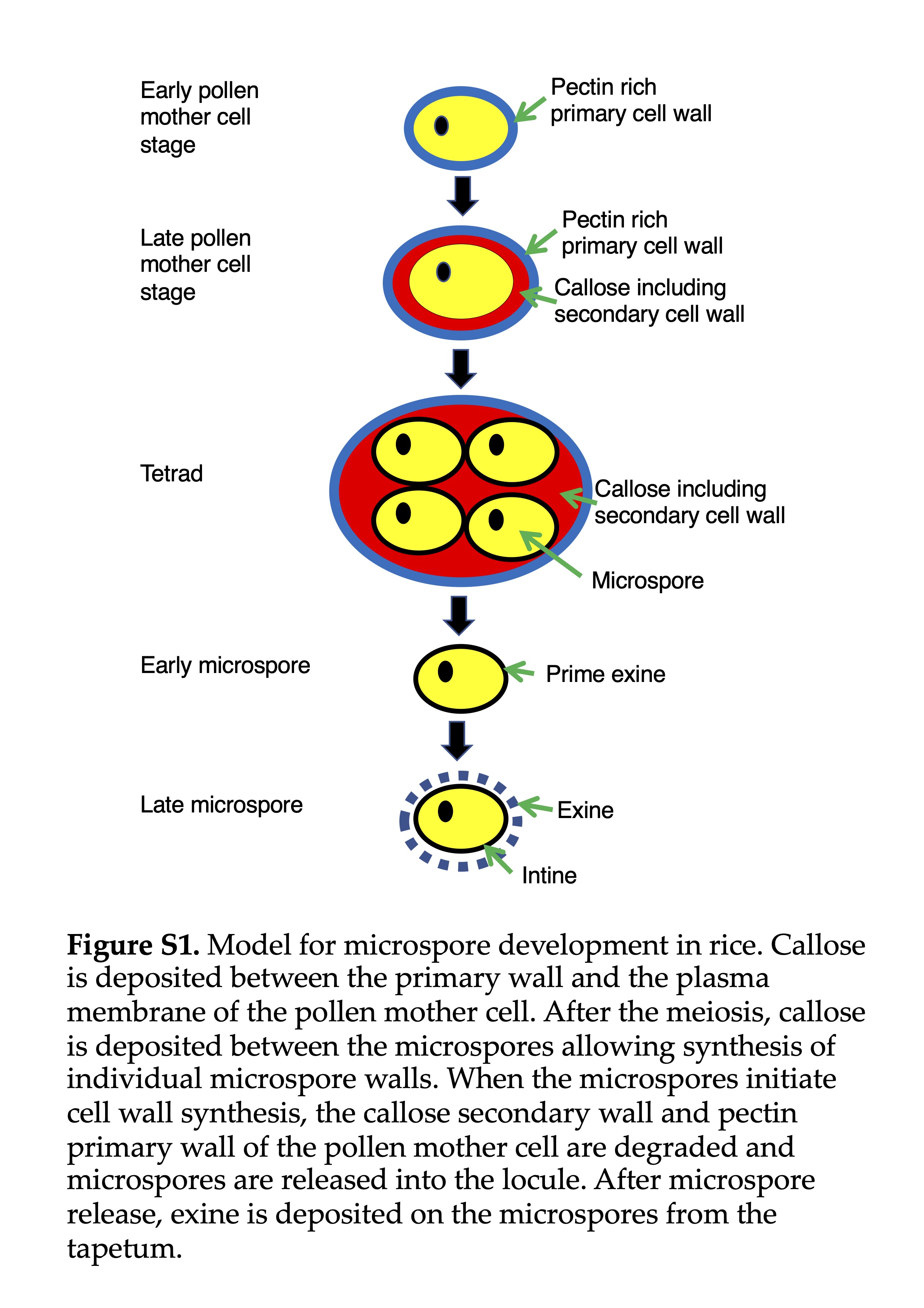

Supplement: Supplementary file 1 [file plants-12-01717-s001.zip › plants-2272646-supplementary.jpg]
